# Antiretroviral Treatment of HIV-2 Infection: Available Drugs, Resistance Pathways, and Promising New Compounds

**DOI:** 10.3390/ijms24065905

**Published:** 2023-03-21

**Authors:** Inês Moranguinho, Nuno Taveira, Inês Bártolo

**Affiliations:** 1 Instituto de Investigação do Medicamento (iMed.ULisboa), Faculdade de Farmácia, Universidade de Lisboa, 1649-019 Lisboa, Portugal; 2 Centro de Investigação Interdisciplinar Egas Moniz (CiiEM), Instituto Superior de Ciências da Saúde Egas Moniz, 2829-511 Caparica, Portugal

**Keywords:** HIV-2, HIV-2 treatment, antiretroviral drugs, resistance mutations, resistance pathways

## Abstract

Currently, it is estimated that 1–2 million people worldwide are infected with HIV-2, accounting for 3–5% of the global burden of HIV. The course of HIV-2 infection is longer compared to HIV-1 infection, but without effective antiretroviral therapy (ART), a substantial proportion of infected patients will progress to AIDS and die. Antiretroviral drugs in clinical use were designed for HIV-1 and, unfortunately, some do not work as well, or do not work at all, for HIV-2. This is the case for non-nucleoside reverse transcriptase inhibitors (NNRTIs), the fusion inhibitor enfuvirtide (T-20), most protease inhibitors (PIs), the attachment inhibitor fostemsavir and most broadly neutralizing antibodies. Integrase inhibitors work well against HIV-2 and are included in first-line therapeutic regimens for HIV-2-infected patients. However, rapid emergence of drug resistance and cross-resistance within each drug class dramatically reduces second-line treatment options. New drugs are needed to treat infection with drug-resistant isolates. Here, we review the therapeutic armamentarium available to treat HIV-2-infected patients, as well as promising drugs in development. We also review HIV-2 drug resistance mutations and resistance pathways that develop in HIV-2-infected patients under treatment.

## 1. Introduction

Acquired immunodeficiency syndrome (AIDS) is caused by human immunodeficiency virus type 1 (HIV-1) and type 2 (HIV-2). HIV-2 is largely confined to West Africa and countries with socioeconomic ties to the region, like Portugal and France [1]. Currently, it is estimated that approximately 1–2 million people worldwide are infected with HIV-2, accounting for 3–5% of the global burden of HIV [2,3].

HIV-2 is composed of nine groups termed A to I, of which group A is by far the most disseminated [4,5]. HIV-2 differs markedly from HIV-1 in the infection course, having a much longer asymptomatic phase, slower clinical progression and lower perinatal and sexual transmission rates (reviewed in [6,7]. In contrast to HIV-1-infected patients, plasma viral load is low or undetectable in most HIV-2-infected patients, irrespective of antiretroviral treatment [8,9]. However, levels of integrated proviral DNA are similar in both infections, suggesting that HIV-2 has a higher tendency for latency establishment than HIV-1 [8,10]. Despite the extended course of the infection, without effective antiretroviral therapy (ART), a substantial proportion of HIV-2-infected patients will progress to AIDS and die [11,12]. Therefore, US guidelines recommend the early initiation of treatment in all patients (regardless of CD4+ cell count and VL) [13]. This is not the case in European guidelines, which recommend the initiation of ARV in all symptomatic patients, but in asymptomatic patients, one of the following conditions should be considered: TCD4+ count ≤500 CD4+ cells/μL, CD4+ T-cell decline of more than 30 cells/μL per year for more than three years, repeatedly detectable HIV-2 RNA in plasma, and comorbidities such as chronic HBV infection [14]. Treatment initiation should be delayed in patients who do not meet any of these criteria.

The available antiretroviral drugs (ARVs) were specifically designed for HIV-1 and some do not work as well, or do not work at all, against HIV-2, since these viruses differ in genetic content by almost 50% and there are important structural differences between the targets of these drugs in HIV-1 and HIV-2 [15,16]. HIV-2 is intrinsically resistant to non-nucleoside reverse transcriptase inhibitors (NNRTIs), the fusion inhibitor enfuvirtide (T-20), some protease inhibitors (PIs), and fostemsavir, an attachment inhibitor that binds to the surface envelope glycoprotein (gp120) and prevents virus binding to the CD4 receptor [17,18,19,20]. In addition, to date, there are no randomized clinical trials to provide orientations for the treatment of HIV-2 infection, particularly concerning the right time to start treatment and the choice of first- and second-line regimens. All clinical decisions rely on results from non-randomized trials, small cohort studies and case series [14].

In many places, rapid antibody tests to differentiate infection with HIV-1 or HIV-2 are not available. This may lead to treatment initiation with ART regimens that are ineffective for HIV-2 (i.e., NNRTI-based) [21,22]. The management of ART in HIV-2-infected patients is suboptimal as it relies mostly on in-house viral load assays with varying clinical performances, and the results cannot be directly compared [14,23,24]. Finally, drug resistance to most drug classes emerges more rapidly in HIV-2 compared to HIV-1, and resistance pathways in HIV-2 may differ from HIV-1 [25,26]. New ARVs are needed for HIV-2, and a better use of the currently available ones is imperative if we want them to remain effective.

Here we review the therapeutic armamentarium presently available to treat HIV-2-infected patients, as well as promising drugs in development. We also identify drug resistance mutations and resistance pathways that develop in HIV-2-infected patients under treatment.

## 2. Reverse Transcriptase Inhibitors

**Figure 1:**
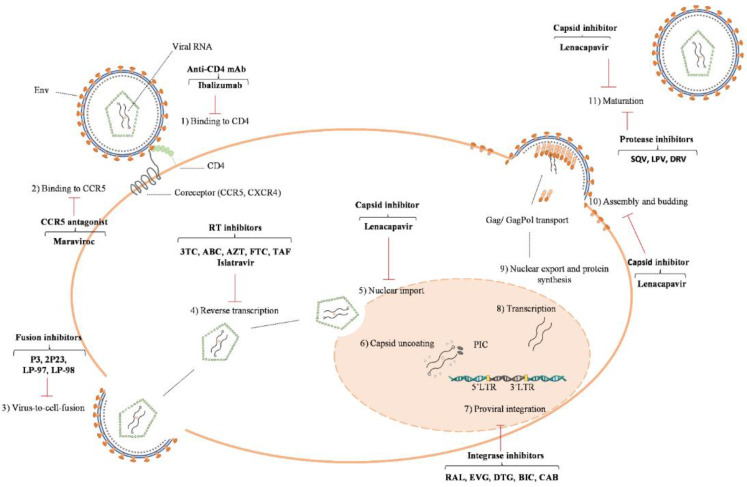
The HIV-2 life cycle and targets for drug intervention. HIV-2 infection begins with the binding of envelope glycoprotein (Env) spikes to receptors on the surface of a CD4 cell (step 1) and to a membrane-spanning co-receptor (e.g., CCR5) (step 2). This process can be disrupted by binding (CD4 binding antibody, Ibalizumab) and entry inhibitors (CCR5 antagonist, Maraviroc). Fusion of the viral and cellular membranes allows the viral particle to enter the cell (step 3). Fusion inhibitors (helical domain 1 binding peptides such as P3, 2P3, LP-97 and LP-98) can be used to block viral fusion and entry. Inside the cell, HIV reverse transcriptase converts genomic RNA into DNA, which is released into the nucleus within the pre-integration complex (PIC) following nuclear import of the capsid (step 5) and capsid uncoating (step 6). The reverse transcription process can be blocked using reverse transcriptase inhibitors (3TC, ABC, AZT, FTC, TDF, TAF). Once in the nucleus, HIV DNA is integrated into the cell’s genome using the enzyme integrase (step 7). HIV DNA integration can be inhibited using integrase strand transfer inhibitors (RAL, EVG, DTG, BIC, CAB). New HIV mRNA is transcribed (step 8) and transported into the cell cytosol (step 9). The mRNA serves as a template for HIV protein synthesis (Gag/Gag-Pol polyproteins, envelope glycoproteins, accessory proteins) and as full-length genomic RNA. These molecules are transported to the cell surface to be incorporated into new viral particles. Viral particle budding, release and capsid maturation are the final steps in the HIV life cycle (steps 10 and 11). The HIV protease enzyme cleaves the Gag/Gag-Pol polyproteins, resulting in the mature capsid and fully infectious viruses. Protease inhibitors (DRV, LPV and SQV) bind to the HIV-2 protease enzyme and interfere with the maturation step of the virus. Lenacapavir is a capsid inhibitor that acts at multiple steps in the HIV replication cycle. The sites of action of clinical inhibitors (red line) are shown.

The reverse transcriptase (RT) enzyme is responsible for the synthesis of proviral DNA, using viral RNA as template (Figure 1). RT has two distinct functions: DNA polymerase that mediates DNA polymerization from DNA and RNA templates, and RNase H that mediates RNA degradation in the context of a DNA/RNA hybrid [27]. HIV-2 RT is a heterodimer composed of two subunits, p68 and p54 [16]. The larger subunit, p68, contains polymerase and RNase H active sites and is divided into four domains: palm, fingers, thumb, connection, and RNase H domains. The smaller subunit, p54, is derived from proteolytic cleavage of p68 by the PR and lacks the RNase H active site [28].

Although sharing a significant amino acid sequence homology, the ATP binding pocket lacks physical depth and is less defined in HIV-2 relative to HIV-1, which may influence the ability to bind ATP [16,27,29]. Furthermore, RT from HIV-2 seems to have reduced processivity, particularly in the presence of low dNTP concentrations [30,31,32]. In this regard, Lenzi et al. [33] have shown that RTs of several HIV-2 strains require higher dNTP concentrations for efficient DNA synthesis compared to HIV-1 RT; this can be relevant for viral replication kinetics in monocytes, macrophages, dendritic cells, and resting CD4+ T cells that have very low dNTP concentrations due to the activity of sterile alpha motif and histidine-aspartate domain containing protein 1 (SAMHD1), a triphosphohydrolase that cleaves dNTPs [34]. The accessory lentiviral protein X (Vpx) from HIV-2 and related SIV lineages degrades SAMHD1 and the human silencing hub (HUSH) complex, and may help to overcome the lower processivity of HIV-2 RT in these cells and increase viral replication [34,35,36]. 

Since proviral DNA synthesis is essential in the HIV replicative cycle, RT has been one of the main targets of ARVs. Currently, there are two RT inhibitor classes, the non-nucleoside (NNRTIs) and the nucleoside/nucleotide RT inhibitors (NRTIs). NNRTIs act by binding in a non-competitive way to HIV-1 RT in an allosteric site approximately 10 Å from the polymerase active site in the palm domain of the p68 subunit, and can induce conformational changes in RT or interfere with the mobility of the enzyme [16,37]. NNRTIs comprise the first-generation inhibitors—delavirdine (DLV), nevirapine (NVP) and efavirenz (EFV), and the second-generation inhibitors—etravirine (ETV), rilpivirine (RPV), doravirine (DOR), and dapivirine (DPV), which is only used in vaginal rings to prevent HIV-1 infection [38,39,40]. The first-generation NNRTIs inhibited HIV-2 strains ROD and EHO at concentrations 50-fold higher than those required to inhibit HIV-1IIIB [41]. Consistent with this, one study showed that treatment of HIV-2 patients with regimens containing NNRTIs results in poor CD4+ T cell recovery relative to HIV-1-infected patients [22]. The resistance of HIV-2 to NNRTIs is due to sequence and structural differences in the pocket binding site at positions 101, 106, 138, 181, 188 and 190 in the RT that may lead to unfavorable contacts with NNRTIs or pocket binding site destabilization [16,42]. Therefore, NNRTIs are not recommended for the treatment of HIV-2 infection.

NRTIs were the first class of ARVs approved by the Food and Drug Administration (FDA) for the treatment of HIV infection. NRTIs, in the triphosphate form, compete with the natural substrates (dTTP, dCTP, dATP or dGTP) of RT [43]. NRTIs lack the 3′OH group that is present in natural RT substrates, and consequently these inhibitors act as obligatory chain terminators, blocking the DNA synthesis.

NRTIs approved for clinical use include lamivudine (3TC), abacavir (ABC), zidovudine (AZT), stavudine (d4T), emtricitabine (FTC), didanosine (ddI), tenofovir (TDF), and tenofovir alafenamide (TAF) [38,40]. Except for d4T and ddI, which are not recommended for the treatment of HIV-2 infection due to the rapid selection of drug resistance [44], NRTIs are the backbone of commonly prescribed regimens against HIV-2. These regimens mainly consist of two NRTIs associated to one integrase strand transfer inhibitor (INSTIs); alternative regimens comprise a boosted PI that is active against HIV-2 (darunavir, saquinavir or lopinavir) plus two NRTIs [14,45,46]. It is noteworthy that the degradation of SAMHD1 by HIV-2 Vpx leads to increased cell concentration of thymidine analogs (AZT and d4T) and may lead to decreased activity of AZT against HIV-2 isolates at clinical concentrations used for HIV-1 [47,48].

TAF is the most recent NRTI in clinical use. It is a prodrug of tenofovir, which is more stable in blood and plasma [49], has more favorable renal and bone safety profiles [50,51], and has higher anti-HIV-1 activity, in oral doses in humans which are ten times lower [52] than TDF. Two studies have shown that TAF is active against clinical isolates from drug naïve and drug-experienced HIV-2-infected individuals with a mean EC_50_ similar to that of HIV-1 [53,54]. Consistent with this, a TAF-based regimen (TAF + FTC + DTG) has been used successfully in men infected with HIV-1 and an HIV-2 isolate that developed resistance to all NRTIs and PIs [55].

Azvudine (FNC) is a novel cytidine analogue with potent activity against HIV-1 (EC_50_ ranging from 0.03 to 6.92 nM) and HIV-2 (EC_50_ ranging from 0.018 to 0.025 nM), and it shows synergism in combination with six approved ARVs [40,56]. FNC potently inhibits some NRTI-resistant HIV-1 strains bearing the L74V and T69N mutations, and M184I appears to be the key resistance mutation selected by this drug [56]. A phase II clinical trial (NCT04109183) has evaluated the safety and efficacy of FNC in HIV-1 infected patients, but its results have not been published so far. Clinical studies of this drug in HIV-2-infected patients have not been reported.

BMS-986001 (also known as festinavir, censavudine and OBP-601), a NRTI structurally related to d4T, exhibits more potent activity against HIV-2 than against HIV-1 in culture [54,57,58]. Festinavir retains full or partial activity against HIV-2 variants with resistance mutations K65R, Q151M, and M184V [54,58]. However, the use of this drug was discontinued due to the rapid acquisition of resistance by HIV-1 and increases in both peripheral and central fat accumulation [59]. Clinical studies have not been undertaken in HIV-2-infected patients.

Islatravir, known as MK-8591 or EFdA (40-ethynyl-2-fluoro-20-deoxyadenosine), is a deoxyadenosine nucleoside analogue that has a very potent activity against HIV-1 and HIV-2 isolates in cell culture, including isolates with pan resistance to other NRTIs [40]. Its mechanism of action differs from the other NRTIs because it retains an extendable 3′OH-group. Islatravir is an RT translocation inhibitor and its incorporation by the RT leads to immediate arrest of polymerization or termination after the incorporation of the next complementary nucleotide [60]. A randomized phase 2b clinical trial of islatravir in combination with doravirine and lamivudine for treatment-naive adults with HIV-1 infection showed, at 48 weeks, a reduction in viral load to lower than 50 HIV-1 RNA copies per mL in up to 90% of the patients receiving the lowest dose (0.25 mg) [61]. The use of this drug for the treatment and prevention of HIV was discontinued in 2021 due to safety concerns about decreases in total lymphocyte count and CD4 count in some participants receiving islatravir in trials. New studies include a clinical trial evaluating the efficacy of a weekly oral combination regimen of islatravir and lenacapavir (NCT05052996) and another evaluating once-daily oral islatravir plus doravirine as initial ART and as switch therapy (NCT04223778). Islatravir has not been used in HIV-2-infected patients.

### HIV-2 Resistance to NRTIs

HIV-1 resistance to NRTIs may evolve by two distinct pathways: (i) exclusion mechanism, in which the mutated RT (main mutations: K65R, K70E, L74V, Q151M and M184I/V) discriminates against the incorporation of the drugs, 3TC, FTC and TDF [62], or (ii) excision ATP-dependent mechanism through the removal of incorporated inhibitors (d4T and AZT) from DNA primers, allowing DNA elongation (thymidine analog-associated mutations—TAMs: M41L, D67N, K70R, L210W, T215Y/F and K219Q/E) [63]. Unlike HIV-1, HIV-2 rarely develops resistance to NRTIs through the excision pathway [32] because the presence of a methionine at position 73 in HIV-2 RT, instead of lysine as found in HIV-1, prevents the development of resistance through the accumulation of TAMs [64]. In HIV-2, nine resistance mutations (K40R, A62V, K65R, K70R, Y115F, Q151M, M184I/V, S215Y) cause significant resistance to 3TC, ABC, AZT and TDF (Table 1) [14,26,65,66,67,68,69,70,71,72,73,74,75].

M184I or M184V are the primary resistance mutations selected under FTC or 3TC treatment, in vitro and in vivo [14,54,74,75,76,77], and lead to a 60-fold increase in IC_50_ for 3TC and 7-fold increase for ABC, compared with wild type HIV-2ROD [67]. Phenotypic studies using recombinant HIV-2 isolates have shown that Q151M has a small impact on the viral susceptibility to 3TC and TDF, confers moderate- to low-level resistance to ABC, and FTC, but is associated with a high level of resistance to AZT [67,70,74]. The Q151M resistance mutation impairs the RT capacity to discriminate between NRTI triphosphates and dNTPs at the nucleotide-binding site [78]. Mutation K65R arises in HIV-2-infected patients exposed to TDF and other NRTIs [14,25,65,75,79] and can be selected in vitro after HIV-2 exposure to increasing doses of TDF [76], and produces a two- to seven-fold decrease in viral susceptibility to the drug [70,76]. Polymorphic mutation V111I increases the fitness of viruses with mutations K65R and Q151M in HIV-2-infected patients [70].

Using site-directed mutagenesis of a HIV-2ROD molecular clone, it was shown that high levels of resistance to festinavir requires the combination of Q151M and M184V and that K65R causes hypersusceptibility to this drug [58]. More recently, high-level resistance to festinavir was reported in a primary isolate with mutations Q151M and K223R [54]. In this study, one isolate with moderate resistance to festinavir had the mutations V111I, Y115F, Q151M, and M184V.

Overall, these studies show that HIV-2 and HIV-1 develop distinct resistance pathways to this drug class. While in HIV-1 the TAMs D67N, K70R, L201W, T215Y/F and K219Q/E are typically selected by NRTIs, in HIV-2 they are rarely selected and M184I/V, K65R and Q151M resistance pathways are preferred.

## 3. Protease Inhibitors

The HIV protease (PR) is an important target in the treatment of HIV infection due to its role in the proteolytic processing of the precursor Gag and Gag-Pol polyproteins during virus maturation, leading to the formation of mature, infection-competent virions [80]. PR has two identical monomers with 99 amino acids and consists of three main domains: the active site (which includes the conserved motif Asp-Thr-Gly), the dimerization domain and the flap region [81]. In the active dimeric state, it forms a central cavity where the natural substrates, the precursor Gag and Gag-Pol polyproteins, bind [82]. Each monomer contributes one Asp25 residue in the catalytic site of the enzyme.

The PR of HIV-1 and HIV-2 have only 39–48% homology at the amino acid sequence level [83]. However, in structural terms, they are very similar, especially in the region of Asp catalytic residues (positions 23–30), as well in a small region that supports catalytic amino acids in an appropriate conformation for catalysis (residues 86–88) [15]. The substrate binding pockets are formed by residues in positions 8, 23, 25, 27–30, 32, 47–50, 53, 76, 80–82 and 84. Of these, only residues in positions 32, 46, 47, 76, 82 differ in both viruses [26,84,85]. Most differences between the PRs are at surface level, while the regions essential for the enzyme function are conserved; however, certain polymorphisms decrease binding affinity of PR for certain inhibitors, and may lead to resistance to PIs [86].

Nine PIs are currently approved for clinical use: first generation—saquinavir (SQV), indinavir (IDV), nelfinavir (NFV), amprenavir (APV), fosamprenavir (FPV) and ritonavir (RTV), used only to boost all PIs except NFV, and second generation—lopinavir (LPV), atazanavir (ATZ), tipranavir (TPV) and darunavir (DRV) [38,80,86]. PIs were designed to bind to the PR active site with higher affinity than the natural substrates and fill more space inside the active site cavity. Resistance mutations in PR lead to an expansion of the active site cavity, thereby reducing PI affinity to that region [87,88,89]. Second generation PIs were designed to surpass HIV-1 resistance to first generation PIs and to improve bioavailability, dosing frequency, and minimize side effects.

Despite the structural similarities, HIV-1 and HIV-2 proteases show major disparities in susceptibility to PIs. LPV, DRV, and SQV are the most potent HIV-2 inhibitors while ATZ, NFV, APV and TPV demonstrate lower potency against HIV-2 and its use is not recommended [14,84,86,90,91,92,93,94].

### HIV-2 Resistance to PIs

HIV-1 resistance to PIs occurs through amino acid substitutions in the binding pocket of the PR or in a nearby site, and reduces the binding affinity of the PIs, ultimately resulting in failure in PR blocking [87,88,89]. Resistance can also occur through the emergence of mutations in Gag-PR cleavage sites [95]. In contrast with HIV-1, little is known about the role of resistance mutations in HIV-2 PR and their effects on viral replication.

HIV-2 resistance pathways to PIs differ from those observed in HIV-1, with V33I, K45R, V47A, I50V, I54M, T56V, V62A, A73G, I82F, I84V, F85L and L90M being the most important HIV-2 PI resistance mutations (Table 1) [26,96]. I54M is the mutation most frequently observed in HIV-2-infected patients treated with PIs [71,75,93,94,96,97,98,99]. In phenotypic assays, this mutation confers high level resistance to LPV with a more than 10-fold increase in IC_50_ compared to wild type virus [100]. In HIV-1, this mutation only occurs under selective pressure with APV [101]. I82F confers high level resistance to LPV (36.3-fold increase) in HIV-2 clinical isolates [100]. The acquisition of the I54M and I82F mutations may lead to cross-resistance to multiple PIs, including LPV [93]. The combination of resistance mutation V62A with natural polymorphism L99F confers high-level resistance to LPV (124-fold) [100]. In the reference HIV-2ROD isolate, the L90M mutation, which is a major resistance mutation to SQV and LPV in HIV-1 [102], conferred resistance to SQV but did not alter the susceptibility to LPV [103]. This mutation has been found in HIV-2-infected patients treated with SQV, IDV, RTV or NFV [75,97,98,104,105,106], frequently in association with other mutations such as I54L/M, V71L, I82F or I84V [97,106]. V47A is frequently found in HIV-2 patients failing LPV-based treatment [71,75,93,94,96,107]. K45R and I64V were also identified in HIV-2 individuals failing treatment with this inhibitor [93]. In vitro, the V47A mutation confers a more than 10-fold resistance to LPV and promotes cross-resistance to IDV, NFV and AMP [108]. However, this mutation causes hypersusceptibility to SQV [93,107]. For that reason, LPV is recommended in first-line therapy and SQV in patients that fail a LPV-based regimen [44]. This mutation causes no resistance to RTV, NFV, TPV and DRV [107,108]. Other mutations such as K7R, V62A/T and L99F are often present in HIV-2-infected patients treated with PIs but are rarely selected in HIV-1 infection [94,97]. K7R was found in HIV-2 patients treated with RTV, LPV or SQV, while L99F was found in patients treated with NFV, LPV, SQV or IDV [106].

In summary, HIV-2 is intrinsically resistant to many PIs, and HIV-1 and HIV-2 show different pathways of resistance to this drug class. Genotypic drug resistance analysis is important when choosing the appropriate second line PI for HIV-2-infected patients failing a PI-based regimen.

## 4. Integrase Strand Transfer Inhibitors

IN is derived from the Gag-Pol polyprotein precursor and catalyzes the integration of the provirus into the host cellular DNA through two catalytic actions: 3′ processing and strand transfer [109]. Each IN monomer consists of three different domains: the N-terminal domain (NTD, HIV residues 1–49), catalytic core domain (CCD, residues 50–212), and C-terminal domain (CTD, residues 213–288) [109]. The CCD domain contains a conserved motif, catalytic triad (64D, 116D and 152E), which is crucial for the catalytic activity of IN. 

The IN proteins of HIV-1 and HIV-2 share the same structure with 65% identity at the amino acid level [110]. Positions involved in the zinc binding domain, the CCD and the DNA binding domain, all crucial to enzyme function, are 100% conserved in HIV-2 [110,111,112]. IN residues in positions that are essential to DNA binding (Q148), integration and replication (Q62, H67, N120, N144, Q148 and N155) in HIV-1 are also conserved in HIV-2 [111].

Integrase strand transfer inhibitors (INSTIs) inhibit the strand transfer step carried out by INs by binding to the active site of the enzyme and also by binding to divalent metals such as Mg^2+^ required for IN catalytic reactions, thus competing with residues located in the CCD [113,114]. This process affects catalytic activity of IN and inhibits the joining of viral and cellular DNA (strand transfer reaction) [113,115,116]. Five INSTIs were approved for clinical use: the first generation raltegravir (RAL) and elvitegravir (EVG), and second generation dolutegravir (DTG), bictegravir (BIC) and cabotegravir (CAB) [38].

RAL was the first INSTI approved by the FDA for clinical use in HIV-1 infection in 2007 and is given once daily (1200 mg) or twice daily (400 mg) in combination with two NRTIs [113,117]. Soon afterwards, Roquebert et al. demonstrated that RAL was a potent inhibitor of HIV-2 clinical isolates (IC_50_ = 1.3–5 nM) [111]. Damond et al. quantified the virological and immunological response to an ARV regimen containing RAL in two patients infected with HIV-2 with multiple drug resistances, high viral load (>6000 HIV-2 RNA copies/mL) and low T CD4+ counts (<25 cells/µL) [118]. Viral load was undetectable after the second month of therapy and significant increases in T CD4+ cell counts were observed and maintained for six months. More recently, in a phase 2 non-comparative trial, 87% of drug-naïve HIV-2-infected patients treated with a RAL-based regimen achieved, at week 48, a viral load < 5 copies/mL and 40% of the patients gained > 100 CD4 T cells/µL [119]. Similar results were obtained in Spain, where undetectable viraemia was achieved in 78% of treatment-naïve patients, with only two patients selecting the N155H resistance mutation [120]. In this study, undetectable viraemia was also observed in 37% of ART-experienced patients, and 80% of patients presenting virological failure exhibited drug resistance mutations. Overall, these reports showed that RAL was effective in suppressing viral load in drug naïve and most treatment experienced HIV-2-infected patients. However, they also showed that RAL-resistance mutations emerge rapidly.

EVG was approved for HIV-1 treatment in 2014 in a fixed-dose combination (Stribild, cobicistat-boosted EVG + FTC + TDF) given once daily [121]. In 2014, Zheng et al. also reported the successful use of Stribild in a drug-naive HIV-2-infected patient [122]. This was consistent with the potent activity of EVG in cell culture against a reference isolate (EC_50_ = 1.6 nM) [76] and clinical isolates from INSTI-naive patients (IC_50_ = 0.3–0.9 nM) [111]. A recent non-randomized clinical trial in Senegal showed that a once-daily, single-tablet-regimen of Stribild is safe, effective, and well tolerated by HIV-2-infected patients [123]. In this study, 28 of the 29 patients (93.3%) who completed the 48 weeks follow-up had viral suppression. Virologic failure occurred in one patient with a multidrug-resistant virus.

DTG, approved in 2013, has potent antiviral activity at 50 mg twice daily in various combination regimens and shows limited cross-resistance to most RAL-resistant HIV mutants [124,125,126], which is likely due to a slower dissociation rate from mutant DNA relative to RAL and EVG, and an ability to adjust its conformation in response to structural changes in the active site of IN [127,128]. Several studies showed that DTG is active against HIV-2 isolates from INI-naïve patients with an IC_50_ range similar to that of HIV-1 [129,130,131]. Consistent with this, a recent retrospective observational study from India showed that most (86%) treatment-naive HIV-2-infected patients receiving a DTG-based regimen achieved an undetectable viral load [132].

Descamps et al. studied 13 HIV-2-infected adults failing a RAL-based therapy who received DTG 50 mg twice daily [133]. Patients with viruses with mutations Y143C/G/H/R at baseline achieved an undetectable plasma viral load, whereas patients with viruses with mutations in codons 148 or 155 did not, indicating that mutations in these codons impact DTG activity in HIV-2 [133]. In Trevino et al. [134], DTG was prescribed for two patients failing a RAL-based therapy due to the N155H mutation, and both experienced a decrease in plasma viral load and an increase in the number of CD4+ T cells. However, viral rebound occurred in one patient after six months, indicating that N155H also affected DTG effectiveness. 

CAB, approved in 2021, is the first long-acting injectable INSTI to be used in combination with RPV as a substitute antiretroviral regimen in HIV-1-infected adult patients who are virologically suppressed [135]. Two studies have evaluated the activity of CAB against HIV-2 isolates in cell culture. In the first study, performed with four isolates, Yoshinaga et al. reported a mean EC_50_ of 0.12 nM [136]. In the second, authors reported an average EC_50_ of 1.8 ± 1.0 nM for group A isolates and 2.6 ± 1.3 nM for group B isolates [137]. These results suggest that CAB in combination with a second drug may be useful in the treatment of HIV-2 infection.

BIC 50 mg daily is given as a fixed-dose combination with FTC and TAF for the treatment of HIV-1 infection [138]. Different cell culture studies have shown that BIC is generally active against HIV-2 group A strains with IC_50_ in the nanomolar range, and also displays potent activity against group B and AB recombinant isolates [131,139,140,141]. Mean instantaneous inhibitory potential (IIP) value of BIC at Cmax was similar to the other INSTIs and was not significantly affected by resistance mutations, highlighting the potential of this drug to treat HIV-2 infection. Clinical studies with BIC are therefore warranted.

### HIV-2 Resistance to INSTIs

A recent meta-analysis showed that Q91R, E92A/Q, T97A, G140S, Y143G, Q148R, A153G, N155H, H156R and an R231 5-amino acid insertion are the most frequent treatment-selected nonpolymorphic mutations in the integrase (Table 1) [26]. As mentioned above, mutations Y143C, Q148K/R and N155H, with or without additional mutations (i.e., E92Q, T97A, G140S), have been associated with failure to most RAL- and EVG-based regimens [26,110,142,143,144,145,146]. Three main mutational pathways confer high-level resistance to RAL and/or EVG: Y143C/G/H/R confers high-level resistance to RAL in combination with E92Q and to EVG in combination with T97A; and G140S/Q148H/K/R and E92Q/N155H confer high-level resistance to both drugs [14,131,145,146,147,148]. This extensive cross-resistance prevents the sequential use of RAL and EVG. 

As for DTG, in reference isolate HIV-2ROD9, single or combinations of mutations E92A, E92Q, E92Q/T97A/A153G and I84V/E92Q/H157S were associated with low level resistance (2.5- to 2.6-fold) [145]. Related with this, one isolate from a patient failing a RAL-based therapy with E92Q and T97A showed intermediate resistance to DTG [131]. Also, the combination of secondary mutations R263K and E92G led to the loss of susceptibility to DTG in HIV-2-infected patients [120]. In HIV-2ROD9, Y143C/G/H/R mutants were susceptible or slightly hypersusceptible to DTG, except for Q91R/E92Q/T97A/Y143G/A153S, which was 7.6-fold resistant to DTG [145]. Mutations at codon 148 alone (Q148K) or in combination with secondary mutations (G140A/S + Q148H/K/R) conferred 5.8- to 370-fold increases in EC_50_. The combination of two primary resistance mutations, Y143H + Q148H or Q148H/R + N155H, resulted in moderate-to-high-level resistance to DTG. Consistent with the latter resistance pattern, patients failing RAL-based treatment due to resistance mutation N155H accumulate variants with mutations in codon 148 (Q148K/R) when treated with DTG [133,149]. Moreover, clinical isolates from RAL-experienced patients with mutations T97A/Y143C have shown a 7-fold increase in EC_50_ for DTG, with G140S/Q148R a 13-fold increase and with G140T/Q148R/N155H a 18-fold increase [130]. Overall, these results suggest that cross-resistance to DTG and RAL is high in HIV-2.

Smith et al. have determined the antiviral activity of CAB against site-directed mutants of HIV-1 and HIV-2ROD9 IN [137]. For HIV-2ROD9, mutants E92Q + Y143C, E92Q + N155H, and G140A + Q148R were 1.5-, 7.5-, and 6.9-fold more resistant to CAB, respectively.

The insertion of five amino-acids at position 231 of the C-terminal domain of HIV-2 IN (231INS), corresponding to a repetition of upstream amino-acids, renders high-level resistance to RAL (from 56 to 150-fold), moderate-to-high-level resistance to EVG (from 8 to 149-fold), low-to-moderate-level resistance to DTG (from 3.3 to 13-fold), low-to-high level resistance to CAB (from 5 to 79-fold), and no or low-level resistance to BIC (from 0 to 5.5-fold) [139,145]. Regarding resistance to BIC, a study with HIV-2ROD9 integrase mutants has shown that G140S/Q148R and G140S/Q148H lead to 34- and 110-fold resistance to bictegravir, respectively [140]. More recently, we have shown that BIC activity was unaffected by combined mutations E92Q/T97A and E92A/Q148K that caused resistance to RAL and DTG [131]. The longer dissociation of BIC from integrase-DNA complexes may contribute to the substantial activity of this drug against HIV-2 isolates with resistance mutations to the other INSTIs [150].

## 5. Entry Inhibitors

Three types of entry inhibitors have been approved for the treatment of HIV-1 infection, the fusion inhibitor (T-20), the CCR5 antagonist (maraviroc, MVC) and the CD4-binding monoclonal antibody (ibalizumab) [38,151]. T20 exhibits reduced activity against HIV-2, showing up to 100-fold higher IC_50_ values in vitro relative to HIV-1 [152], and is not recommended for HIV-2 infection. Other fusion inhibitor peptides are being studied and some have shown promising results. Peptide P3, an ancestral peptide derived from helical region 2 in the transmembrane glycoproteins of HIV-2 and SIV [153], potently inhibits both HIV-1 and HIV-2 cell entry and replication (IC_50_ of 63.8 nM for HIV-2 and 11 nM for HIV-1) [153]. It was not possible to select P3-resistant isolates in cell culture, suggesting a high genetic barrier to resistance [153]. 2P23 is another promising short-peptide fusion inhibitor derived from HIV-1, HIV-2 and SIV [154]. This peptide was able to successfully inhibit HIV-1 isolates, T20-resistant HIV-1 mutants, and a panel of primary HIV-2 isolates, HIV-2 mutants and SIV isolates. More recently, Xue et al. characterized two cholesterol modified peptides derived from the transmembrane envelope region, LP-97 and LP-98, as extremely potent inhibitors of HIV-1, HIV-2, SIV and SHIV isolates in vitro, ex vivo and in vivo in Rhesus monkeys [155]. Comparing to T20, AZT and 3TC, LP-97 and LP-98 were more potent and broader HIV-1 and HIV-2 inhibitors with impressive IC_50_ values (~0.03 to 0.07 pM). LP-80 is currently in clinical trials (NCT04592315) to access the safety, tolerability and pharmacokinetics in HIV-1-infected patients without prior antiviral therapy.

MVC binds to a CCR5 cavity within the two, three, six and seven transmembrane helices (reviewed in [152]). MVC binding induces conformational changes in the second extracellular loop of CCR5, preventing interaction with the surface envelope glycoprotein gp120 of HIV. MVC is only active against viruses with CCR5 tropism, and a tropism test is mandatory before starting therapy with this inhibitor. There is ample data on the activity of MVC against HIV-1 in cell culture, and MVC has been successfully used in the treatment of HIV-1 infection [156,157,158]. In contrast, there is limited data on the phenotypic susceptibility of HIV-2 to MVC in vitro, and the use of this drug in HIV-2 infection remains to be explored. Three studies have shown that the potency of MVC against HIV-2 isolates in cell culture is similar to HIV-1, with IC_50_ values between 0.175 to 2.1 nM [159,160,161]. However, Borrego et al. demonstrated that MVC inhibits R5 HIV-2 strains with higher IC_90_ values (IC_90_ = 42.7 nM) than those obtained for HIV-1 (IC_90_ = 9.7 nM), and also that inhibition of HIV-2 R5 viruses from AIDS patients requires higher MVC concentrations than R5 viruses from early infections [159]. Consequently, higher doses of this compound may be necessary in the treatment of HIV-2 infection.

So far, only two clinical studies have reported the use of MVC in the salvage therapy of HIV-2 patients with limited therapeutic options [162,163]. It was demonstrated that both regimens, MVC and RAL or MVC and Foscarnet, increased the T CD4+ cell count and decreased the viral load to undetectable levels [162,163]. However, these clinical cases do not provide valuable information about the relative efficacy of MVC as salvage therapy in HIV-2-infected patients, since MVC was used in combination with other antiretroviral drugs and a genotypic or phenotypic tropism test was not performed before treatment. In this regard, it should be noted that there is a genotypic online tool to determine HIV-2 coreceptor usage based on the V3 envelope sequence [164].

Ibalizumab, a long-acting monoclonal antibody, is a CD4-directed post-attachment inhibitor approved for the treatment of infection with multidrug-resistant HIV-1 strains [151]. Le Hingrat et al. recently evaluated the susceptibility of sixteen group A and B HIV-2 primary isolates to Ibalizumab [165]. Results showed an inhibition of all 16 isolates with a median IC_50_ value of 0.027 µg/mL, which is in the range of IC_50_ observed for HIV-1. This data encourages the use of Ibalizumab in patients with multidrug-resistant HIV-2.

## 6. Lenacapavir

Lenacapavir (LEN), formerly GS-6207, is a recently FDA-approved, first-in-class, long-acting capsid (CA) inhibitor for the treatment of heavily treatment-experienced adults with multidrug-resistant HIV-1 isolates [166,167]. Lenacapavir can be administered subcutaneously up to every six months, minimizing pill burden and potentially improving adherence [166]. It binds to the hydrophobic CA pocket formed by two adjacent CA subunits within the hexamer, stabilizing the CA core and preventing the binding of cellular cofactors (CPSF6 and Nup153) that aid viral nuclear import and integration of HIV [168,169]. Lenacapavir also interferes with Gag/Gag-Pol function and reduces the production of capsid protein subunits [169]. In cell culture, it has potent HIV-1 antiviral activity in the low picomolar range (EC50 = 50–314 pM) [168,169].

In vitro resistance to LEN is associated with variants in the CA portion of Gag (L56I, M66I, Q67H, K70N, N74D/S and T107N) [167]. These mutations appear to occur very rarely (<1%) in drug-naive patients [170], in isolates from treatment-experienced patients [171] and in isolates with resistance mutations to the other drug classes [172].

Only one study has evaluated the activity of lenacapavir against HIV-2. Link et al. showed that GS-6207 was active against two HIV-2 isolates (CBL20 and CDC310319) in human peripheral blood mononuclear cells, but less so compared to HIV-1 (mean EC50 = 885 pM) [167]. More data are needed on the antiviral activity of lenacapavir against primary isolates of HIV-2. There are no ongoing or planned clinical trials of lenacapavir in HIV-2-infected patients.

## 7. Spiro-β-Lactams Are Potent Inhibitors of HIV-2 Infection

Spiro-β-lactams are a new class of compounds with potent (nM) activity against HIV-1 (mean IC_50_ of the lead compound BSS-730A = 14 nM) [173,174]. More recently, we have shown that BSS-730A also has potent activity against primary HIV-2 isolates from INI-naïve patients (mean IC_50_ = 18.10 nM) [131]. Moreover, BSS-730A activity was unaffected by integrase mutations that confer HIV-2 resistance to RAL and DTG. The mechanism of action of these compounds is still unknown, but BSS-730A displays strong synergism with RAL and AMD3100, a CXCR4 antagonist [131,174]. These compounds have yet to be tested in the clinic. In this regard, the finding that the IIP value of BSS-730A is higher than that of INIs highlights the potential clinical value of this compound [131].

## 8. Conclusions

Deciding which therapeutic regimens to implement in HIV-2-infected patients is a challenge due to the limited number of effective drugs and the limited number of treatment studies and clinical trials. An additional problem is the low genetic barrier to resistance of HIV-2, the limited understanding of resistant pathways, and the frequent cross-resistance between drugs from the same class. Investigational drugs like azvudine, islatravir and BMS-986001, new generation fusion inhibitor peptides such as P3, 2P23, lipopeptides LP-97 and LP-98 and spiro-β-lactam BSS-730A are active against HIV-2 in vitro but in vivo studies are needed to determine the usefulness of these drugs for HIV-2 infection. RAL and DTG show potent activity against HIV-2 and are increasingly being used in first-line treatment regimens. BIC has yet to be used in the clinic but its activity profile against RAL- and DTG-resistant isolates indicates that it should be useful for patients failing therapy with these drugs. MVC appears to be an interesting therapeutic option for R5 isolates, but its use is limited by the lack of clinical studies and the high cost. Clinical studies are needed with Ibalizumab for the treatment of infection by multi-drug-resistant HIV-2 isolates. Given the aim of curing and eradicating HIV infection as a public health problem by 2030, studies on the activity of antiretroviral drugs in HIV-2 replication in cell reservoirs such as macrophages and dendritic cells are urgently needed.

## Figures and Tables

**Table 1:** Antiretroviral drugs active against HIV-2 and key mutations causing resistance to drugs in clinical use.

Drug Class	Name	Development Phase	Resistance Mutations
Nucleoside Reverse Transcriptase Inhibitors	Lamivudine	Clinical use	K65R, K70E, L74V, Q151M, M184I/V
	Abacavir	Clinical use	
	Zidovudine	Clinical use	
	Emtricitabine	Clinical use	
	Tenofovir	Clinical use	
	Tenofovir alafenamide	Clinical use	
	Azvudine	Investigational	not determined
	Festinavir	Investigational	Q151M and M184V or K223R
	Islatravir	Investigational	not determined
Protease Inhibitors	Lopinavir	Clinical use	V33I, K45R, V47A, I50V, I54M, T56V, V62A, A73G, I82F, I84V, F85L, L90M
	Darunavir	Clinical use	
	Saquinavir	Clinical use	
Integrase Strand Transfer Inhibitors	Raltegravir	Clinical use	Q91R, E92A/Q, T97A, G140S, Y143G, Q148R, A153G, N155H, H156R, five amino acid insertions in R231
	Elvitegravir	Clinical use	
	Dolutegravir	Clinical use	
	Bictegravir	Clinical use *	
	Cabotegravir	Clinical use *	
Fusion Inhibitors	P3	Investigational	not determined
	2P23	Investigational	not determined
	Lipopeptides (LP97; LP98)	Investigational	not determined
	Ibalizumab	Clinical use *	not determined
CCR5 Antagonist	Maraviroc	Clinical use	not determined
Capsid Inhibitor	Lenacapavir (LEN)	Clinical use *	not determined
Other	Spiro-β-lactams	Investigational	not determined

* Only approved for HIV-1-infected patients.

## Data Availability

The data presented in this study are available in this article.
